# Supplementary material for: A Short ERAP2 That Binds IRAP Is Expressed in Macrophages Independently of Gene Variation
Source: Int J Mol Sci. 2022 Apr 29;23(9):4961. doi: 10.3390/ijms23094961 (PMC9101739; doi:10.3390/ijms23094961)
Supplement: Supplementary file 1 [file ijms-23-04961-s001.zip › ijms-1666647-supplementary.pdf]

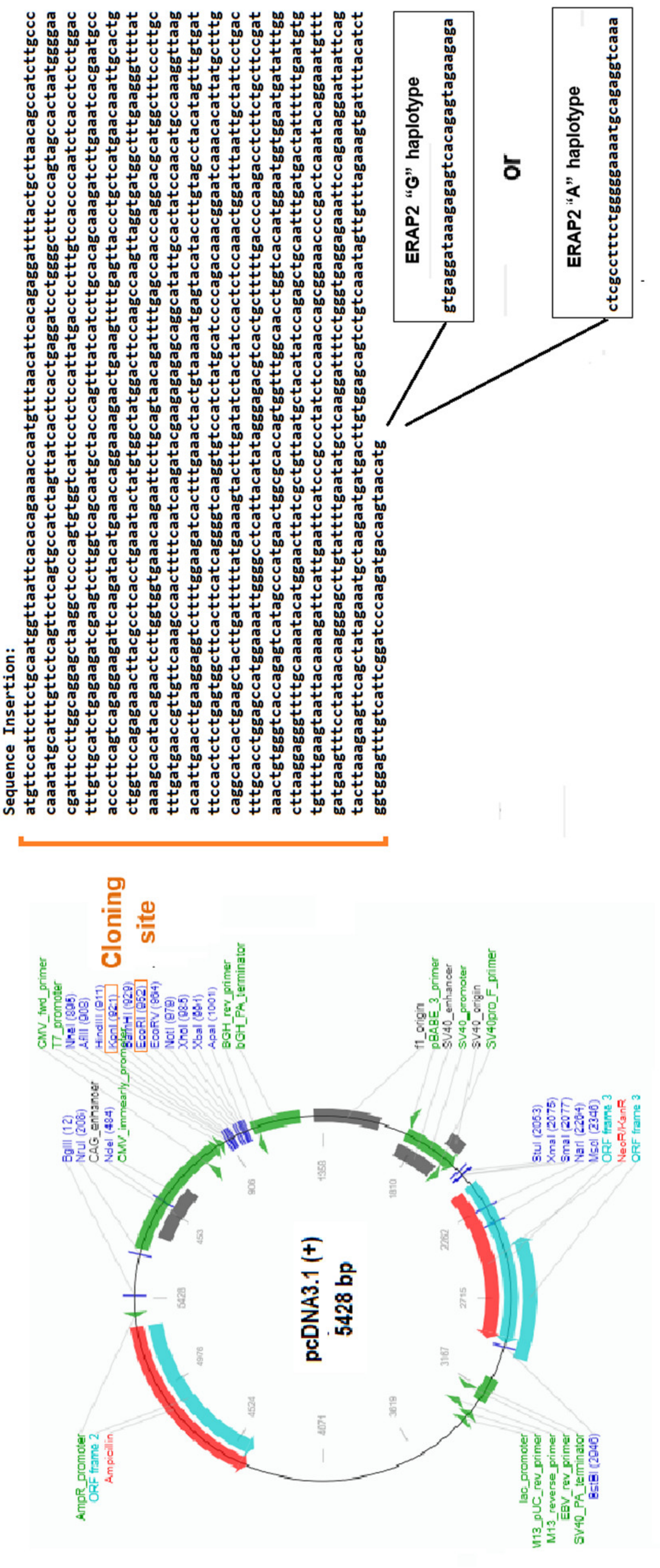

Figure S1. Detailed DNA sequences of the constructs carrying the ERAP2 derived from the "G" or the "A" haplotype encoding the first 532 or 534 amino acids respectively. The two sequences differ at the C-terminus one transcribing for the natural "G" mRNA (VRIKRVTE) and the other for the alternative "A" mRNA but interrupted at the same length as the "G" haplotype (LAFLGENAEVK)

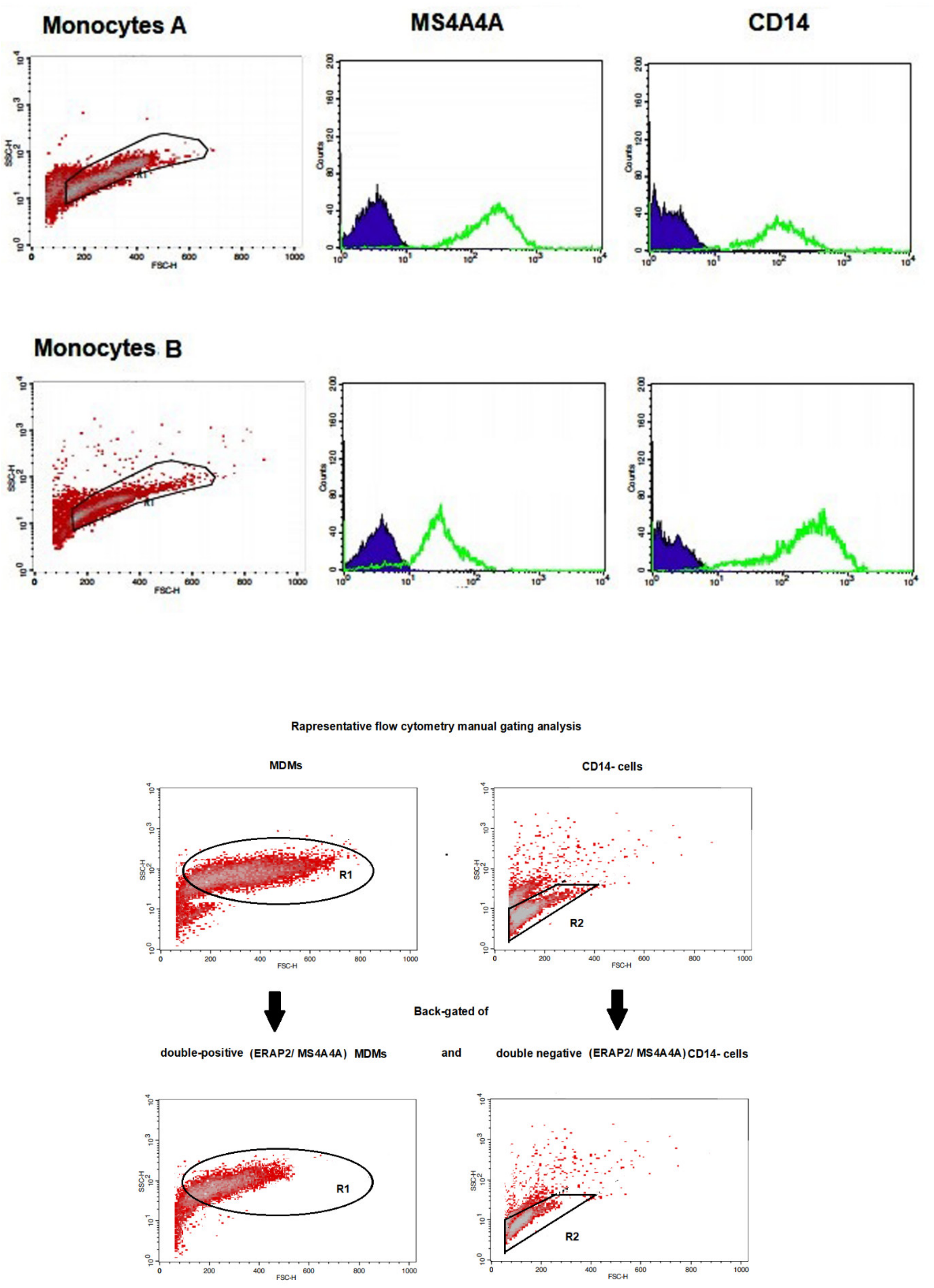

Figure S2. Monocyte/macrophages cytofluorimetric analysis. Upper panel: Representative analysis of the expression of MS4A4A and CD14 in monocytes genotyped as A/A (A) or G/G (B) at rs2248374. **Lower panel**: gating of the double positive ERAP2/MS4A4A in CD14+ (left) or CD14 neg (right) PBMCs.

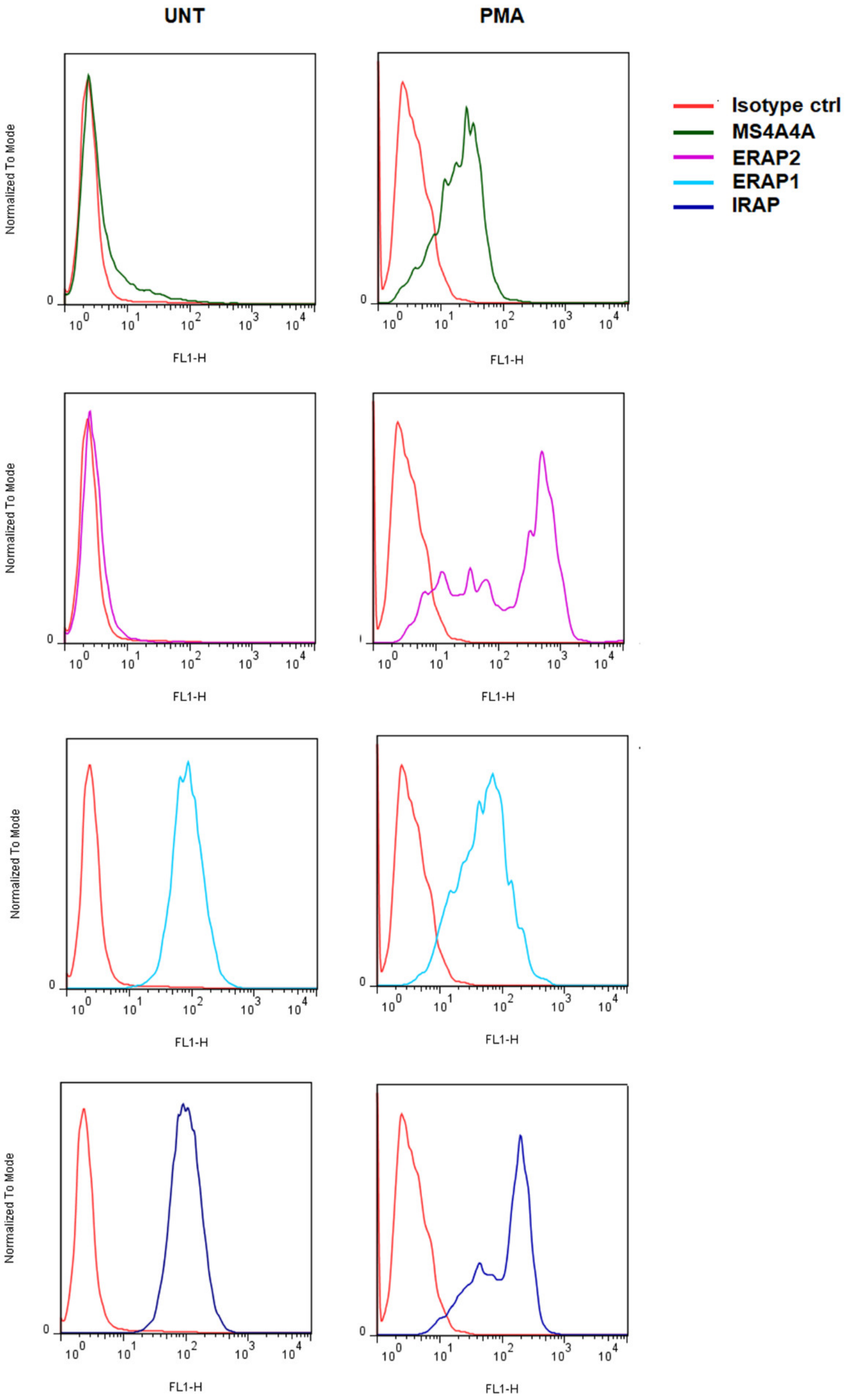

Figure S3. U937 surface expression of MS4A4A, ERAP2, ERAP1 and IRAP. Cytofluorimetric analysis of the expression of MS4A4A (upper panels), ERAP2 (second panels), ERAP1 (third panels) and IRAP in untreated (lower panels) (left panels) and PMA-treated (right panels) U937 cells.

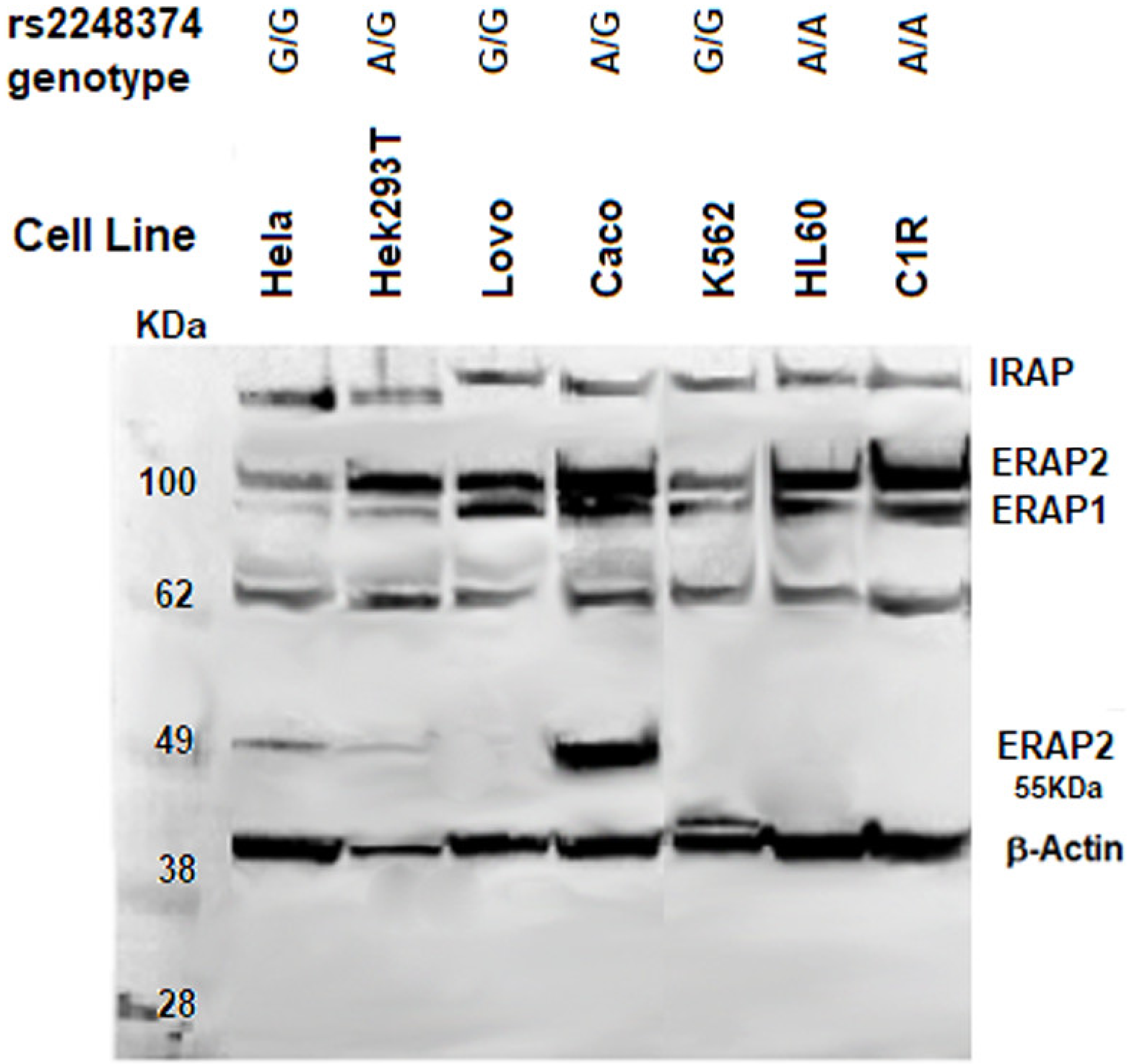

Figure S4. Expression of ERAP2, ERAP1 and IRAP in selected cancer cell lines genotyped for the SNP rs2248374. The respective rs2248374 genotypes are indicated at the top of the image.
